# Assessing Habitat Suitability and Climate-Change Responses of Raptors in Hunan Province, China, Using Ensemble Species Distribution Models

**DOI:** 10.3390/ani16111722

**Published:** 2026-06-04

**Authors:** Yufeng Zeng, Zongze Zhou, Hao Meng, Zeshuai Deng, Wen Peng, Daode Yang

**Affiliations:** 1 Institute of Wildlife Conservation, College of Forestry, Central South University of Forestry and Technology, Changsha 410004, China; 20231100117@csuft.edu.cn (Y.Z.); 18909452005@163.com (H.M.); 20210100016@csuft.edu.cn (Z.D.); cavendish1731@163.com (W.P.); 2 Hunan Provincial Forestry Bureau, Changsha 410399, China; hnlyrjc@163.com

**Keywords:** raptors, species distribution models, biomod2, habitat suitability, climate change, environmental drivers, Hunan Province

## Abstract

Raptors are high-trophic-level predators and scavengers that are sensitive to habitat alteration, human disturbance, and climate variability, yet province-wide assessments of their habitat suitability and climate-change responses remain limited in subtropical China. Hunan Province, located along the inland section of the East Asian–Australasian Flyway, contains complex mountain systems, plains, wetlands, and land-use mosaics that may support diverse raptor assemblages. Based on raptor survey records collected across Hunan from January 2022 to July 2023, we used biomod2 ensemble species distribution models to assess current habitat suitability, identify key environmental predictors, and project future changes under the SSP2-4.5 and SSP5-8.5 scenarios for the 2050s and 2090s. We recorded 39 raptor species and retained 3637 valid geographic locations and 4855 observed individuals after data cleaning. Nine representative species were further selected to construct 22 species–season combinations covering resident species, summer visitors, winter visitors, and four phenological stages. The EMwmean weighted ensemble model consistently outperformed the best single models, increasing mean AUC from 0.882 to 0.970 and the mean TSS from 0.611 to 0.845. Temperature seasonality (BIO4), the Human Footprint Index (HFP), precipitation in the driest month (BIO14), and the Normalized Difference Vegetation Index (NDVI) were the dominant predictors, although their relative importance varied among residency types and phenological stages. Under current conditions, highly suitable and most suitable habitats covered 65,259.67 km^2^, accounting for 30.81% of Hunan Province, and were mainly concentrated in western, southern, and eastern mountain regions. Future projections indicated a marked contraction of high-suitability habitats, especially under SSP5-8.5, with no HSI > 0.6 habitat identified by the 2090s. High-suitability habitats also became increasingly concentrated at higher elevations. These findings identify mountain regions as key conservation priorities and provide a spatial framework for climate-adaptive raptor conservation in Hunan Province.

## Simple Summary

Raptors, such as hawks, eagles, falcons, and owls, play important roles in ecosystems but are vulnerable to habitat change, human disturbance, and climate change. Hunan Province in central–southern China contains mountains, hills, plains, and wetlands, providing diverse habitats for many raptor species. In this study, we used field survey records from 2022 to 2023 and ecological modeling methods to identify areas that are currently suitable for raptors and to estimate how these areas may change under future climate scenarios. We recorded 39 raptor species and modeled the habitat suitability of nine representative species across different seasons. Highly suitable habitats were mainly located in the western, southern, and eastern mountain regions of Hunan. However, future climate projections suggested that these highly suitable habitats may shrink substantially, especially in the high-emission scenario, by the late 21st century. The remaining suitable areas also tended to become more concentrated at higher elevations. These findings can help identify priority areas for raptor conservation and support climate-adaptive biodiversity management in Hunan Province.

## 1. Introduction

Raptors occupy high trophic levels as predators and scavengers in terrestrial ecosystems, where they contribute to food-web regulation and ecosystem stability [1,2,3]. Their large home ranges, low population densities, and sensitivity to habitat alteration, human disturbance, and climate variability make their distributions useful indicators of environmental change [4]. Land-use change, urban expansion, agricultural intensification, infrastructure development, and climate warming have increasingly altered habitat availability and seasonal resource dynamics [5,6,7]. Identifying suitable habitats and environmental drivers is therefore important for understanding raptor spatial ecology and supporting regional conservation planning [8,9].

Habitat suitability assessments are widely used to examine species distributions and inform biodiversity conservation. For raptors, previous studies have combined remote sensing, geographic information systems, and species distribution models to assess climatic, topographic and anthropogenic drivers [10,11,12,13,14,15]. However, many assessments still focus on single species, local areas, or static time periods. This limits their ability to capture variation among raptor assemblages, residency types and phenological stages. Resident species, summer visitors, and winter visitors may differ in habitat requirements across breeding, migration and wintering periods [16,17,18].

Species distribution models (SDMs) link occurrence records with environmental predictors to estimate suitable habitats across space and time [19,20,21]. Individual algorithms, including regression-based, machine learning and maximum-entropy approaches [22,23,24,25], differ in assumptions and sensitivity to data structure [26,27]. This can increase uncertainty when modeling species with broad ranges, sparse records or seasonal movements. Ensemble SDMs address part of this uncertainty by integrating predictions from multiple algorithms and weighting them by predictive performance [28,29,30]. The biomod2 platform provides a flexible framework for ensemble modeling in habitat suitability assessments and climate-change projections.

Hunan Province in central–southern China provides a useful regional system for assessing raptor habitat suitability because its subtropical mountains, plains, wetlands and land-use mosaics create strong environmental gradients. It also lies along an inland section of the East Asian–Australasian Flyway, potentially supporting seasonal movements by migratory raptors. Although historical records indicate diverse raptor fauna, previous works mainly focused on species inventories, distribution records and local observations. A province-wide, multi-species and phenology-aware assessment of raptor habitat suitability, environmental drivers and climate-change responses remains lacking.

To address this gap, we combined field-based raptor occurrence records with multi-source environmental predictors and used the biomod2 to develop ensemble SDMs for raptors in Hunan Province. We aimed to (1) evaluate the performance of ensemble models in predicting suitable habitats for representative raptor species and species–season combinations; (2) identify the main environmental predictors of habitat suitability and compare their relative importance among residency types and phenological stages; and (3) project changes in suitable habitats and elevational distributions under future climate scenarios. By integrating multi-species occurrence data, seasonal classification, ensemble modeling, and climate-scenario projections, this study provides a spatially explicit basis for identifying priority areas and informing climate-adaptive conservation strategies for raptors in subtropical China.

## 2. Materials and Methods

### 2.1. Study Area

**Figure 1:**
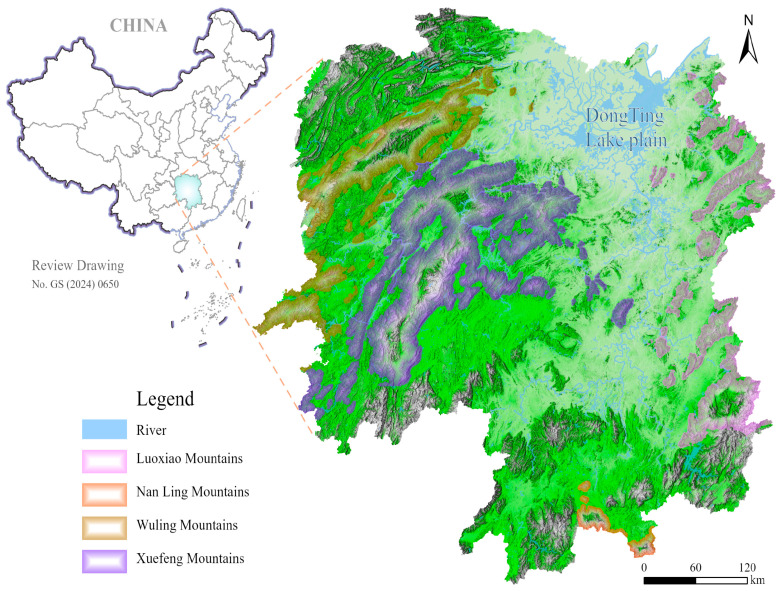
Location, topographic pattern, and major mountain ranges of the study area in Hunan Province.

Hunan Province is located in central–southern China, extending from 108°47′ to 114°15′ E and 24°38′ to 30°08′ N, with an area of approximately 2.12 × 10^5^ km^2^. The province has a horseshoe-shaped topography, with the Wuling and Xuefeng Mountains in the west, the Nanling Mountains in the south, the Luoxiao Mountains in the east, and the Dongting Lake Plain in the north (Figure 1). Elevation ranges from approximately 20 to 2110 m.

Hunan has a humid mid-subtropical monsoon climate, with a mean annual temperature of 16–18 °C and annual precipitation of 1200–1500 mm. The combined effects of complex terrain, climatic gradients, wetlands, forests, farmlands, and other land-cover types provide diverse habitat conditions for raptors. The province is also located along an inland section of the East Asian–Australasian Flyway, potentially supporting migration, stopover, foraging, and seasonal habitat use by raptors.

### 2.2. Raptor Occurrence Data

#### 2.2.1. Data Sources and Phenological Classification

Raptor occurrence records were obtained from province-wide field surveys conducted in Hunan Province from January 2022 to July 2023. The surveys were designed to cover the main geographical regions and habitat types of the province, including mountain areas, forest margins, wetlands, farmlands, river valleys, reservoirs, and other open or semi-open habitats where raptors are likely to occur. Survey routes were arranged across different parts of Hunan Province to improve spatial representativeness.

Field surveys were primarily conducted using line transects and supplemented by point-count surveys at suitable vantage points. During line-transect surveys, observers recorded raptors detected visually or acoustically along survey routes. Point-count surveys were used mainly in open areas, wetlands, reservoirs, mountain valleys, and other sites where raptors could be observed from fixed positions. Surveys were conducted under suitable weather conditions whenever possible, avoiding periods of heavy rain, dense fog, or very poor visibility.

For each raptor record, species identity, number of individuals, geographic coordinates, date, survey location, and habitat information were recorded. Species identification was based on direct field observation using binoculars and cameras, together with morphological characters, flight behavior, calls, and field-guide references. Records with uncertain species identification, obvious coordinate errors, duplicate information, or locations outside Hunan Province were removed during data cleaning. The dataset included raptors belonging to Accipitriformes, Falconiformes, and Strigiformes, with a total of 39 species recorded in the study area.

#### 2.2.2. Data Cleaning, Spatial Thinning, and Species Selection

Occurrence records were screened to remove invalid entries, including erroneous coordinates, uncertain species identifications, and locations outside Hunan Province. After cleaning, 3637 valid geographic locations representing 4855 observed individuals were retained for subsequent modeling.

To reduce spatial clustering and sampling bias, records were spatially thinned separately for each species–season combination using the R version 4.4.3 package spThin. A thinning distance of 10 km was applied, considering the spatial scale of province-wide modeling and the high mobility of most raptors, ensuring a minimum great-circle distance of 10 km between retained records [26,31,32].

Species–season combinations were selected for modeling based on sample size, spatial representativeness, seasonal coverage, residency type, and ecological interpretability. Although 39 raptor species were recorded in the field surveys, not all species were suitable for species distribution modeling. Many species had too few valid occurrence records after data cleaning and spatial thinning, and some were recorded only occasionally, during limited seasons, or as passage migrants or vagrant species. Such sparse or seasonally incomplete records may lead to unstable model calibration, overfitting, and unreliable spatial projections.

Therefore, only species–season combinations with at least 20 valid occurrence records after spatial thinning were retained for modeling. In addition, we considered whether the retained combinations could represent different residency types and phenological stages. This screening procedure resulted in nine representative species and 22 species–season combinations covering resident species, summer visitors, winter visitors, and four phenological stages. The remaining recorded species were retained for describing the overall raptor assemblage in Hunan Province but were not included in SDM analyses because they did not meet the minimum data requirements for robust model fitting.

### 2.3. Environmental Predictors and Preprocessing

#### 2.3.1. Predictor Sources and Selection

We compiled an initial predictor set representing climate, topography, vegetation, land-use and human disturbance. The predictors included 19 bioclimatic variables, elevation, the Normalized Difference Vegetation Index (NDVI), land-use type, and the Human Footprint Index (HFP). Bioclimatic variables and elevation were obtained from WorldClim 2.1 at 30 arc-second resolution, approximately 1 km. NDVI was derived from the 2022 NASA MODIS MOD13A3 vegetation index product. Land-use data from the 30 m land-use product released by the Resource and Environment Science and Data Center of the Chinese Academy of Sciences. HFP data were obtained from the global Human Footprint dataset used in this study [33].

To ensure spatial consistency, all raster layers were harmonized using the WorldClim 2.1 bioclimatic layers as the spatial reference. Continuous predictors, including elevation, NDVI and HFP, were resampled to 1 km using bilinear interpolation. The categorical land-use layer was resampled using the nearest-neighbor method to preserve class attributes. All predictors were projected to the same coordinate reference system, aligned to the same grid, and clipped to the boundary of Hunan Province.

The 2022 MOD13A3 NDVI layer was used as a static predictor for all seasonal models to represent broad-scale vegetation greenness and cover. Land-use type (LANDUSE) retained its original class codes and was not converted to a factor variable. Therefore, LANDUSE was interpreted only in terms of relative variable importance rather than as an ordinal response The HFP layer was retained at its original value scale before model fitting.

To reduce multicollinearity and improve model stability, we first used Pearson correlation analysis to remove one variable from each highly correlated pair when |r| > 0.7. Variance inflation factor (VIF) analysis was applied to further assess predictor redundancy and exclude variables with VIF > 10. Seven predictors were retained for modeling: temperature seasonality (BIO4), minimum temperature of the coldest month (BIO6), precipitation of the driest month (BIO14), precipitation seasonality (BIO15), NDVI, LANDUSE and HFP. Elevation was included in the initial predictor set but was excluded from the final models after collinearity screening. The final predictor set was used for current habitat suitability modeling and future climate-scenario projections.

#### 2.3.2. Future Climate Scenarios

Future climate data were obtained from the WorldClim 2.1 CMIP6 dataset. To reduce structural uncertainty associated with a single global climate model, we selected three general circulation models: BCC-CSM2-MR, CNRM-CM6-1, and EC-Earth3-Veg. Two shared socioeconomic pathway scenarios were used: SSP2-4.5, representing an intermediate-emission pathway and SSP5-8.5, representing a high-emission pathway. Future projections were conducted for the 2050s (2041–2060) and the 2090s (2081–2100).

Given the uncertainty in long-term projections of land use, vegetation dynamics and human disturbance, NDVI, LANDUSE, and HFP were held constant in future projections. Only the bioclimatic predictors were replaced by their corresponding future values. Based on three GCMs, two SSP scenarios, and two future periods, 12 future environmental predictor stacks were constructed for spatial projection of the ensemble models.

For each SSP–period combination, HSI rasters were generated separately for the three GCMs and then averaged pixel by pixel. This produced the final prediction for each scenario and period. Subsequent spatial visualization and area estimation were based on four multi-GCM averaged future scenarios: SSP2-4.5 2050s, SSP5-8.5 2050s, SSP2-4.5 2090s and SSP5-8.5 2090s.

### 2.4. Species Distribution Modeling

#### 2.4.1. Model Calibration and Evaluation

Because the raptor occurrence data consisted mainly of presence records and reliable true-absence data were unavailable, pseudo-absence points were generated for SDM calibration. The entire area of Hunan Province was used as the candidate background region. Grid cells at 1 km resolution that contained known occurrence records were excluded to avoid overlap between presences and pseudo-absences. Pseudo-absence points were generated independently for each species–season combination.

Pseudo-absence points were generated using the surface range envelope (SRE) strategy implemented in biomod2. The environmental envelope was defined using the 2.5th and 97.5th percentiles of environmental values at the occurrence points. For each species–season combination, pseudo-absence sampling targeted at least 5000 points and at least five times the number of valid presence records. If fewer candidate cells were available, all available candidate cells were used. Presence and pseudo-absence points were then combined for model calibration and evaluation.

Single-algorithm SDMs were calibrated separately for each species–season combination using the selected environmental predictors in biomod2. Ten algorithms were included: generalized linear models (GLMs), generalized additive models (GAMs), multivariate adaptive regression splines (MARSs), generalized boosting models (GBMs), random forests (RF), artificial neural networks (ANNs), MAXNET, XGBOOST, classification tree analysis (CTA), and surface range envelope (SRE).

Model calibration used a repeated random-split validation strategy with 10 replicate runs (CV.nb.rep = 10). In each run, 80% of the data were used for training and 20% for testing (CV.perc = 0.8). A fixed random seed (seed.val = 2025) was used to ensure reproducibility. Model tuning was performed using the biomod2 option OPT.strategy = “bigboss”.

Model performance was evaluated using the area under the receiver operating characteristic curve (AUC) and the true skill statistic (TSS) [34,35,36]. The TSS was calculated as follows:

(1) TSS=Sensitivity+Specificity−1.

Because the number of presence records varied among species–season combinations and large pseudo-absence samples may reduce sample prevalence, the TSS was used as the primary criterion for model screening and ensemble selection, and the AUC was used as a complementary metric. The means and standard deviations of the AUC and TSS across replicate runs were calculated to summarize model accuracy and stability. Variable importance was estimated three times for each model using a random permutation. The change in model predictions after permuting each predictor was used to quantify its relative importance.

#### 2.4.2. Ensemble Modeling and Spatial Projection

To reduce algorithm-specific uncertainty and improve prediction stability, ensemble models were constructed from calibrated single-algorithm models. Single models were selected for ensemble construction based primarily on the TSS. Models with TSS ≥ 0.7 were preferentially retained; when no model reached this threshold for a given species–season combination, the threshold was relaxed, but only models with TSS > 0 were included.

Three ensemble statistics were calculated: simple mean ensemble prediction (EMmean), coefficient of variation among ensemble predictions (EMcv), and weighted mean ensemble prediction (EMwmean). EMwmean was used for the final habitat suitability projections. Model weights were assigned using proportional decay (EMwmean.decay = “proportional”), allowing better-performing single models to contribute more strongly to the ensemble prediction.

The final ensemble models were projected onto the environmental space of Hunan Province under current and future climate scenarios at 1 km resolution. The EMwmean prediction probability produced by biomod2 was used as the habitat suitability index (HSI), ranging from 0 to 1. Higher values indicated greater habitat suitability. The administrative boundary of Hunan Province was used as a spatial mask to remove values outside the study area.

For each species–season combination, HSI rasters were generated for current conditions and future scenarios. After HSI rasters were standardized to the same coordinate reference system, spatial resolution, and study-area extent. Rasters from all species–season combinations were averaged pixel by pixel with equal weights to produce an integrated HSI raster for each scenario.

### 2.5. Habitat Suitability and Elevational Change Analysis

Integrated HSI rasters were classified into five suitability levels using consistent thresholds: unsuitable habitat, 0–0.2; low-suitability habitat, 0.2–0.4; moderately suitable habitat, 0.4–0.6; highly suitable habitat, 0.6–0.8; and most suitable habitat, >0.8 [37]. The same classification thresholds were applied to current and future HSI rasters, Future classification was based on the multi-GCM averaged HSI rasters for each SSP–period combination. For each scenario, we calculated the area and percentage of each suitability level. We focused particularly on high-suitability habitats, defined as grid cells with HSI > 0.6, to evaluate potential contraction, expansion, and spatial redistribution under future climate scenarios.

To examine elevational redistribution of high-suitability habitats, integrated HSI rasters were overlaid with a digital elevation model (DEM) harmonized to the same coordinate reference system, spatial resolution, and extent as the HSI rasters. For each scenario, elevation statistics were extracted for grid cells with HSI > 0.6. These included the mean elevation, the median elevation, the peak elevation of the kernel density distribution, and main high-density elevational ranges. Kernel density estimation (KDE) was used to generate elevation–density curves for high-suitability habitats under current and future climate scenarios. For future scenarios in with no grid cells with HSI > 0.6, elevation statistics and KDE curves were not calculated.

## 3. Results

### 3.1. Raptor Species Records and Modeled Species–Season Combinations

From January 2022 to July 2023, 39 raptor species belonging to three orders and five families were recorded across Hunan Province, including Accipitriformes, Falconiformes, and Strigiformes. After data cleaning, 3637 valid geographic locations representing 4855 observed individuals were retained. The recorded species included 21 resident species, two summer visitors, eight winter visitors, seven passage migrants, and one vagrant species. All recorded species were listed as nationally protected wildlife in China.

Nine representative species were selected for species distribution modeling, producing 22 species–season combinations across three residency types and four phenological stages. The 39 recorded species were used to describe the overall raptor assemblage in Hunan Province. The nine modeled species represented the subset with sufficient occurrence records and seasonal coverage for SDM calibration. These included four resident species, two summer visitors, and three winter visitors. Among the 22 species–season combinations, five corresponded to spring migration, six to the breeding season, five to autumn migration, and six to the wintering season (Table 1).

### 3.2. Model Performance and Ensemble Model Evaluation

**Figure 2:**
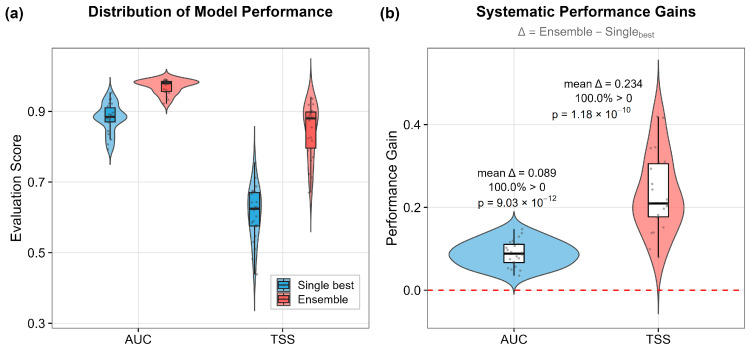
Performance comparison between the best single models and ensemble models across 22 species–season combinations.

Across the 22 species–season combinations, EMwmean weighted ensemble models consistently outperformed the best-performing single models under the same cross-validation framework (Figure 2). The mean AUC increased from 0.882 ± 0.042 for the best single models to 0.970 ± 0.020 for the ensemble models, while the mean TSS increased from 0.611 ± 0.077 to 0.845 ± 0.077. The average improvements were 0.089 ± 0.031 for the AUC and 0.234 ± 0.094 for the TSS.

Improvements in both metrics were positive for all species–season combinations (Figure 2b). Two-sided one-sample *t*-tests showed that increases in the AUC and TSS were significantly greater than zero, with *p* = 9.03 × 10^−12^ and *p* = 1.18 × 10^−10^, respectively. The distribution of the TSS shifted toward higher values in the ensemble models, indicating higher predictive performance and model stability (Figure 2a).

Among single-model algorithms, GBM and MAXNET generally showed relatively high performance and were most frequently selected as the best-performing single models. Subsequent habitat suitability projection, variable-importance assessment, and future scenario projection were conducted using the EMwmean weighted ensemble model.

### 3.3. Environmental Variable Importance and Response Differences

**Figure 3:**
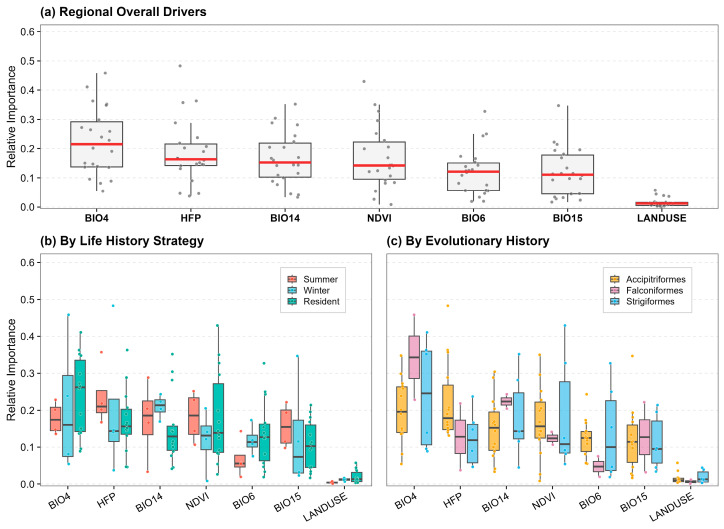
Environmental variable importance for raptor habitat suitability models (box plots showing mean ±95% confidence interval).

The relative importance of the seven environmental predictors varied among the 22 species–season combinations (Figure 3). At the regional scale, temperature seasonality (BIO4) had the highest overall importance, with a median value of 0.215 and a mean ± SD of 0.223 ± 0.114. It was followed by the Human Footprint Index (HFP; median = 0.163), precipitation of the driest month (BIO14; median = 0.152), and the Normalized Difference Vegetation Index (NDVI; median = 0.142). The minimum temperature in the coldest month (BIO6) and precipitation seasonality (BIO15) showed lower overall importance, and LANDUSE had consistently low importance across species–season combinations.

**Figure 4:**
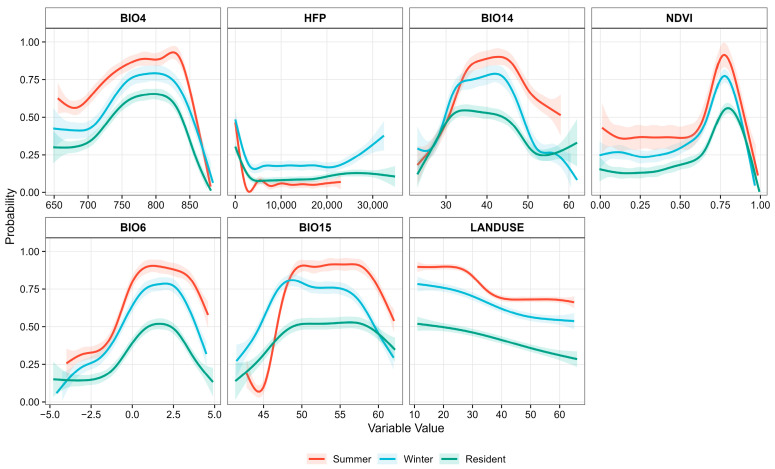
Marginal response curves of habitat suitability to environmental predictors by residency type.

Variable-importance rankings differed among residency types (Figure 3b). For resident species combinations, BIO4 had the highest median relative importance, followed by HFP, NDVI, and BIO14. For summer visitor combinations, HFP ranked highest, while NDVI and BIO14 also showed relatively high importance. For winter visitor combinations, BIO14 was the most important predictor, followed by BIO4 and HFP. Marginal response curves also showed residency-specific differences in environmental responses (Figure 4). Summer visitors showed higher responses at relatively high NDVI values, winter visitors responded strongly to BIO14, and resident species showed a distinct response to BIO6. Response ranges of HFP also differed among residency types.

**Figure 5:**
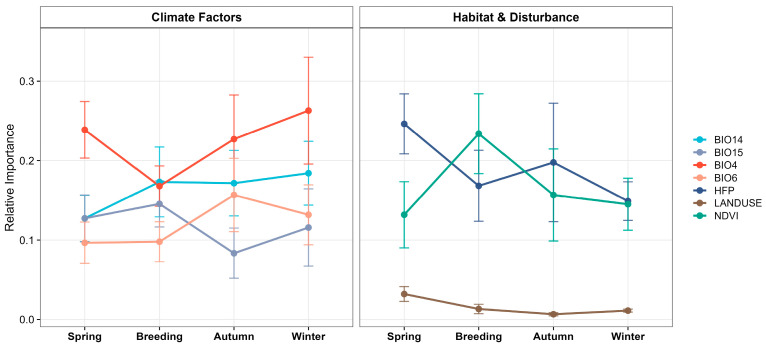
Seasonal dynamics of environmental variable importance in raptor habitat suitability models.

Variable importance also varied among phenological stages (Figure 5). During spring migration, HFP had the highest mean relative importance, followed by BIO4 and NDVI. During the breeding season, NDVI ranked highest, followed by BIO14 and HFP. During autumn migration, BIO4 had the highest importance, followed by HFP and BIO14. During wintering, BIO4 remained the most important predictor, followed by BIO14 and HFP. Across phenological stages, BIO4 was most important during wintering, HFP during spring migration, and NDVI during the breeding season. LANDUSE remained low across all four phenological stages.

Variable-importance rankings also differed among taxonomic groups, although sample sizes were uneven (Figure 3c). BIO4 ranked relatively high across Accipitriformes, Falconiformes, and Strigiformes. HFP showed higher median importance in Accipitriformes, whereas BIO14 ranked relatively high in Falconiformes and Strigiformes.

### 3.4. Current Potential Distribution Patterns

**Figure 6:**
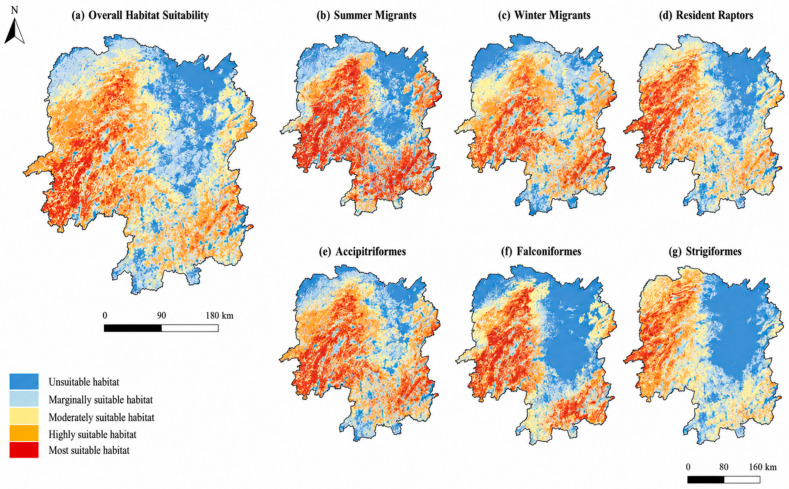
Current potential habitat suitability of raptors in Hunan Province.

The integrated habitat suitability map, obtained by averaging current HSI rasters across the 22 species–season combinations, showed marked spatial heterogeneity in potential raptor habitats in Hunan Province (Figure 6). The mean integrated HSI was 0.450 and the median was 0.443, indicating an overall moderate level of habitat suitability. Highly suitable and the most suitable habitats were mainly distributed in the western, southern, and eastern mountain regions, where they formed patchy or belt-like patterns along major mountain systems. By contrast, the Dongting Lake Plain and some low-elevation areas in central Hunan were dominated by unsuitable and low-suitability habitats.

Based on the five-level HSI classification, moderately suitable habitats accounted for 24.59% of the province. Highly suitable and most suitable habitats accounted for 24.05% and 6.75%, respectively. Together, these two classes covered 65,259.67 km^2^, representing 30.81% of Hunan Province.

Current potential suitable habitats also differed among residency types (Figure 6b–d). Summer visitors had the largest combined proportion of highly suitable and most suitable habitats, accounting for 39.03% of the province. They were followed by winter visitors at 37.08% and resident species at 28.27%. Winter visitors had the highest mean HSI, whereas summer visitors showed the highest proportion of most suitable habitats. Because the number of modeled species and species–season combinations differed among residency types, these comparisons should be interpreted as relative patterns within the modeled dataset rather than as overall differences among all raptors in Hunan Province.

### 3.5. Projected Changes in Suitable Habitats Under Future Climate Scenarios

**Figure 7:**
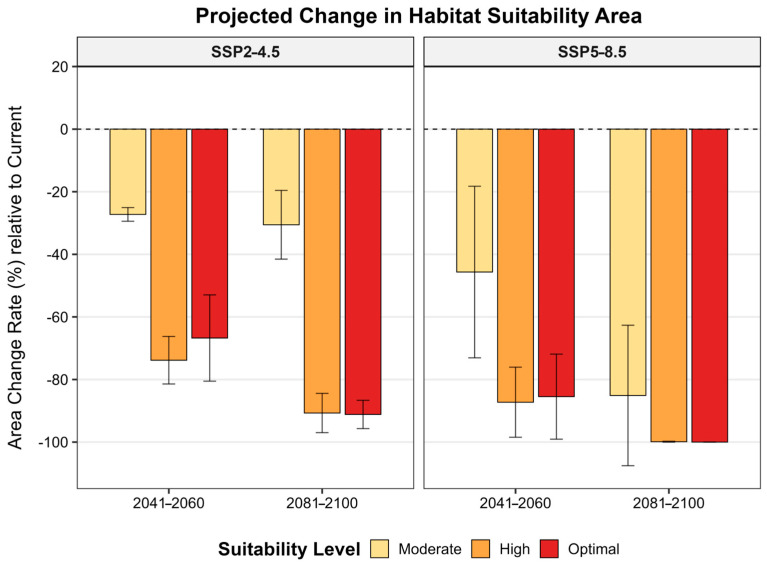
Projected changes in suitable habitat area under future climate scenarios.

**Figure 8:**
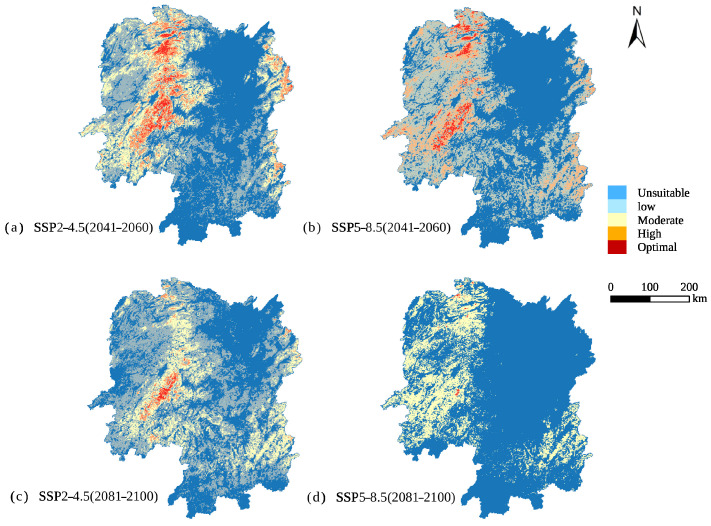
Projected habitat suitability classes of raptors in Hunan Province under future climate scenarios.

Future climate scenarios projected an overall decline in suitable habitats for raptors in Hunan Province, with stronger reductions under the high-emission scenario and in the late-century period (Figure 7 and Figure 8). Moderately suitable, highly suitable, and the most suitable habitats all decreased relative to current conditions, with the largest losses occurring in highly suitable and most suitable habitats.

Under current conditions, highly suitable and most suitable habitats cover 65,259.67 km^2^, accounting for 30.81% of Hunan Province. This area decreased to 16,036.98 km^2^ (7.50%) under SSP2-4.5 in 2041–2060 and to 3537.99 km^2^ (1.65%) under SSP5-8.5 in 2041–2060. In the 2081–2100 projections, highly suitable and most suitable habitats covered only 3738.92 km^2^ (1.75%) under SSP2-4.5, whereas no habitat with HSI > 0.6 was identified under SSP5-8.5. Moderately to most suitable habitats showed a similar declining trend, decreasing from 117,346.85 km^2^ under current conditions to 48,064.11 km^2^ under SSP2-4.5 in 2041–2060, 23,587.46 km^2^ under SSP5-8.5 in 2041–2060, 32,065.20 km^2^ under SSP2-4.5 in 2081–2100, and only 265.60 km^2^ under SSP5-8.5 in 2081–2100.

**Figure 9:**
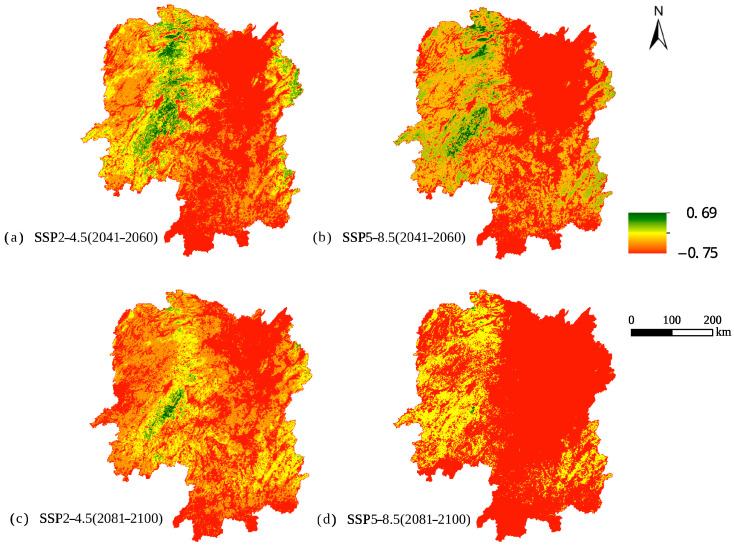
Projected HSI changes in current core suitable habitats.

Spatially, most future highly suitable and most suitable habitats were retained in western Hunan and several local mountain areas. Unsuitable and low-suitability habitats expanded under all future scenarios, most prominently under SSP5-8.5 in 2081–2100. Within the current suitable area (HSI > 0.4), HSI values generally declined under future climate scenarios (Figure 9). This indicates that many areas currently classified as moderately suitable or above were projected to shift toward lower suitability classes, particularly under SSP5-8.5 and in the late-century period.

### 3.6. Elevational Redistribution of Highly Suitable Habitats

**Figure 10:**
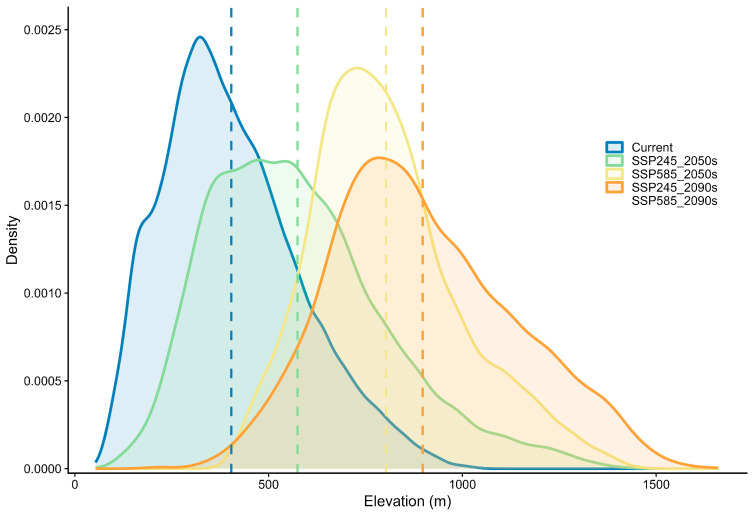
Elevational redistribution of highly suitable raptor habitats under future climate scenarios.

The elevational distribution of highly suitable and most suitable habitats indicated that current high-suitability habitats for raptors in Hunan Province are mainly concentrated in areas with low to middle elevations (Figure 10). Under current conditions, areas with HSI > 0.6 had a mean elevation of 403.81 m and a median elevation of 380.31 m. The peak of the kernel density distribution occurred at approximately 322.19 m, and the main high-density elevational range was 153.29–557.76 m. These results indicate that existing high-suitability habitats are primarily concentrated in areas with elevations below approximately 600 m.

Under future climate scenarios, the elevational distribution of highly suitable and most suitable habitats shifted upward where such habitats remained. Mean elevation increased to 574.72 m under SSP2-4.5 in the 2050s, 803.09 m under SSP5-8.5 in the 2050s, and 897.63 m under SSP2-4.5 in the 2090s. The corresponding KDE peaks also shifted upward, from 322.19 m under current conditions to 473.14 m, 729.62 m, and 783.99 m in the three future scenarios, respectively. These shifts indicated a transition of high-suitability habitats from low-elevation areas toward middle- and higher-elevation zones. Under SSP5-8.5 in the 2090s, no highly suitable or most suitable habitat was identified; therefore, elevation statistics and KDE curves were not calculated for this scenario.

## 4. Discussion

### 4.1. Performance and Applicability of the Ensemble Modeling Framework

The EMwmean ensemble models consistently outperformed the best single models across the 22 species–season combinations, as indicated by higher AUC and TSS values. This performance supports the use of a biomod2-based ensemble modeling framework for estimating potential raptor habitat suitability in Hunan Province. In this environmentally heterogeneous region, individual algorithms may be sensitive to model assumptions, sampling structure and species-specific responses [38]. By integrating multiple algorithms and assigning greater weights to better-performing models, ensemble models can reduce algorithm-specific uncertainty and improve predictive stability [39]. Similar studies have also shown that ensemble SDMs often provide more stable predictions than single-algorithm models, particularly across large regions with strong environmental gradients [40,41].

The higher performance of the EMwmean model supports its use in habitat suitability mapping, environmental predictor assessment, and future climate projections. However, model performance metrics should not be interpreted as evidence of ecological causality. The AUC and TSS evaluate model discrimination under the available occurrence and pseudo-absence data, whereas habitat suitability maps represent model-based estimates of potential environmental suitability. The ensemble framework therefore provides a useful spatial modeling tool, but its outputs should be interpreted alongside field knowledge, species ecology, and the limitations of occurrence-based modeling.

### 4.2. Environmental Drivers of Raptor Habitat Suitability

The relative importance of environmental predictors suggested that raptor habitat suitability in Hunan Province is jointly associated with climatic seasonality, human disturbance, dry-season water availability, and vegetation cover. Temperature seasonality (BIO4), HFP, precipitation in the driest month (BIO14) and NDVI were the main predictors, whereas LANDUSE showed relatively low importance. This pattern suggests that provincial-scale raptor suitability reflects multiple environmental gradients rather than a single dominant habitat factor.

The high importance of BIO4 may reflect the role of seasonal thermal variability in shaping raptor activity, energetic constraints, and seasonal resource availability. Temperature seasonality may also affect raptors indirectly through prey activity, vegetation phenology, and seasonal foraging conditions. The relatively high importance of HFP suggests that human disturbance is an important component of raptor habitat suitability at the provincial scale. Urban expansion, transportation networks, agricultural land use, and other human activities can modify habitat structure. They may also reduce the availability of disturbance-sensitive foraging or nesting areas and alter prey resources. BIO14 and NDVI further indicate that dry-season moisture and vegetation cover may affect raptor suitability by affecting prey availability, cover conditions, stopover environments, and foraging habitats stability [15].

Environmental responses differed among residency types. For resident species, the high importance of BIO4 suggests that species remaining in the same region throughout the annual cycle may be more closely associated with climatic conditions that remain suitable across seasons. Because resident raptors complete breeding, wintering, and other non-migratory activities within the same broad region, their distributions may be more strongly constrained by annual climatic seasonality [42]. For summer visitors, the high importance of HFP, together with NDVI and BIO14, suggests sensitivity to disturbance gradients, vegetation cover, and moisture conditions. For winter visitors, the importance of BIO14 indicates that dry-season precipitation is an important predictor of winter habitat suitability [16]. Under the relatively dry and cool winter conditions of Hunan Province, dry-season moisture may influence prey resources in wetlands, farmlands, forest edges, and open habitats, thereby affecting wintering habitat suitability.

Predictor importance also varied among phenological stages. The high importance of HFP during spring migration suggests that migrating raptors may be sensitive to disturbance gradients in stopover sites, foraging areas, and movement corridors. During the breeding season, NDVI became the most important predictor, indicating that vegetation cover and habitat structure may be relevant to nesting concealment, breeding safety, and local prey availability [43,44]. During autumn migration and wintering, BIO4 remained highly important, while BIO14 increased in importance during wintering. These patterns suggest that non-breeding distributions may be jointly influenced by climatic seasonality and dry-season moisture. Overall, these differences highlight the need to interpret raptor habitat suitability in relation to residency type and phenological stage, rather than using a single season or ecological group to represent provincial-scale raptor responses [45].

### 4.3. Current Spatial Patterns and Ecological Interpretation

The current integrated HSI map indicates that potential suitable habitats for raptors in Hunan Province are closely associated with mountain landscapes. Highly suitable and most suitable habitats are concentrated mainly in the western, southern, and eastern mountain regions, whereas the Dongting Lake Plain and parts of central Hunan are dominated by lower suitability classes. This pattern suggests that mountainous landscapes are important areas for maintaining high-suitability raptor habitats in the province.

The concentration of high-suitability habitats in mountain regions may be related to their strong topographic gradients, diverse vegetation types, high habitat heterogeneity, and lower levels of intensive urban development than in lowland plains. Mountain–forest mosaics, forest edges, valleys, and open patches can provide nesting, foraging, perching, and seasonal movement opportunities for raptors with different ecological requirements. These heterogeneous habitats may also support diverse prey communities and suitable hunting conditions. By contrast, lowland plains and highly modified landscapes are often characterized by greater human disturbance, more intensive agricultural or urban land use and reduced structural heterogeneity. These factors may decrease habitat suitability for some raptor groups.

Differences among residency types should be interpreted cautiously at the assemblage level. Although summer visitors and winter visitors showed larger areas of highly suitable and most suitable habitats than resident species within the modeled dataset, these differences may partly reflect uneven numbers of modeled species and species–season combinations, seasonal habitat use, and occurrence-data structure. They should therefore be interpreted as relative patterns within the selected modeling framework rather than complete differences among all raptors in Hunan Province. Nevertheless, the consistent concentration of suitable habitats in mountain regions suggests that these areas should be considered spatial priorities for raptor conservation and habitat management.

### 4.4. Projected Habitat Contraction Under Future Climate Scenarios

Future climate projections suggested a substantial contraction of suitable raptor habitats in Hunan Province. The strongest losses of highly suitable and most suitable habitats were projected for SSP5-8.5 by the late-century period. No habitat with HSI > 0.6 was identified for SSP5-8.5 in the 2090s. This suggests that high-suitability habitats may be more vulnerable to future climatic change than lower-suitability habitats.

This contraction may reflect the dependence of high-suitability habitats on specific combinations of climatic and habitat conditions. Under current conditions, these habitats are mainly concentrated in the western, southern, and eastern mountain regions, where suitable climatic conditions, relatively high vegetation cover, and lower human disturbance may coincide. Future changes in temperature seasonality, minimum temperature conditions, or dry-season precipitation could reduce the climatic suitability of these areas. As a result, some currently high-suitability habitats may shift towards moderate, low-suitability, or unsuitable classes. Similar ensemble SDM studies have reported contractions or spatial shifts in suitable habitats under future climate scenarios as climatic conditions change [17,46].

The projected reduction in highly suitable habitats should be interpreted as a model-based signal of declining potential suitability or contraction of core suitable environmental space. It should not be interpreted as direct evidence of raptor population decline, local extinction, or disappearance of individuals from these areas. Projected habitat changes primarily reflect the response of environmental suitability to future climatic conditions and do not dynamically incorporate future land-use change, vegetation dynamics, urban expansion, agricultural restructuring, or conservation interventions. Future studies integrating land-use simulations, vegetation dynamics, long-term monitoring, and population-dynamic data would provide a more comprehensive assessment of climate-related risks for raptors in Hunan Province [47,48,49].

### 4.5. Elevational Redistribution and Conservation Implications

Overlaying HIS rasters with elevation showed that current highly suitable and most suitable habitats are mainly distributed at low to middle elevations. Future high-suitability habitats, where retained, became increasingly concentrated at higher elevations and in localized mountain areas. This pattern suggests that climate change may lead not only to habitat contraction but also to a redistribution of suitable environmental space along elevational gradients.

The upward shift may reflect the vertical redistribution of suitable climatic conditions under future climate change [50,51]. In Hunan Province, the Wuling, Xuefeng, Nanling and Luoxiao Mountains contain substantial topographic variation and habitat heterogeneity. These areas may therefore provide potential spatial refugia for some raptor species under future climatic conditions.

From a conservation perspective, the projected contraction and elevational concentration of high-suitability habitats suggest that raptor conservation in Hunan Province should consider mountain regions that retain relatively high suitability under both current and future scenarios [52]. Conservation planning could prioritize habitat quality and landscape connectivity across major mountain regions. Such planning may help maintain seasonal movement, dispersal and access to alternative habitats under changing climatic conditions [53]. Management strategies should also account for differences among resident species, summer visitors, and winter visitors because environmental drivers and suitable habitat patterns differed among residency types and phenological stages [54,55]. Integrating current high-suitability areas, future stable habitats, and potential elevational refugia into regional planning would support more climate-adaptable raptor conservation in Hunan Province.

### 4.6. Limitations and Future Research

Several limitations should be considered when interpreting the results of this study. First, although occurrence records were cleaned and spatially thinned, the dataset may still be influenced by uneven survey effort, accessibility, and residual spatial clustering across a large geographic area. Second, reliable true-absence data were unavailable, and pseudo-absence selection can affect model calibration and projected suitability patterns. Third, future projections held NDVI, LANDUSE, and HFP constant and replaced only the retained bioclimatic variables. This approach allowed us to focus on climate-change effects but did not account for future vegetation dynamics, land-use conversion, urban expansion, agricultural change, or shifts in human disturbance. Finally, the models estimated potential habitat suitability rather than actual abundance, population trends, reproductive success, survival, or occupancy. Species interactions, prey availability, nesting habitat, microclimatic refugia, and disturbance-sensitive behavioral responses were not explicitly included.

Future studies should combine long-term monitoring, telemetry tracking, prey surveys, nesting habitat assessment, dynamic land-use projections, and alternative pseudo-absence or occupancy-modeling approaches to validate and refine the model outputs. Integrating SDMs with population-level data and protected-area gap analyses would further improve the identification of conservation priorities and climate-resilient habitat networks for raptors in Hunan Province.

## 5. Conclusions

This study used a multi-species, multi-season ensemble modeling framework to assess the spatial patterns, environmental drivers and climate-change responses of potential raptor habitats in Hunan Province. The biomod2-based ensemble models showed improved predictive performance and were used to estimate current and future habitat suitability. Raptor habitat suitability was jointly associated with climatic seasonality, human disturbance, dry-season moisture, and vegetation cover, with temperature seasonality showing the highest overall importance.

Under current conditions, highly suitable and most suitable habitats cover 65,259.67 km^2^, accounting for 30.81% of Hunan Province, and are mainly concentrated in the western, southern, and eastern mountain regions. Future climate scenarios projected a marked contraction of suitable habitats, especially highly suitable and most suitable habitats. Under SSP5-8.5 in the 2090s, no habitat with HSI > 0.6 was identified. Where high-suitability habitats remained under other future scenarios, they tended to shift towards higher elevations. These patterns indicate a contraction and elevational redistribution of core suitable environmental space rather than direct evidence of raptor population movement.

Overall, these finding suggest that conservation planning should consider mountain habitats that retain high suitability under both current and future scenarios. Maintaining ecological connectivity among major mountain systems and incorporating potential climate refugia and stable habitats into regional panning may support more climate-adaptive raptor conservation in Hunan Province. Future studies integrating long-term monitoring, dynamic land-use projections, and protected-area coverage analyses would further refine conservation priorities.

## Figures and Tables

**Table 1:** Species-season combinations used for ensemble species distribution modeling.

Species	Residency Type	Spring Migration	Breeding Season	Autumn Migration	Wintering Season
*Accipiter virgatus*	Resident	√	√	√	√
*Spilornis cheela*	Resident	√	√	√	√
*Buteo buteo*	Winter visitor	√		√	√
*Glaucidium brodiei*	Resident	√	√		√
*Glaucidium cuculoides*	Resident		√	√	√
*Aviceda leuphotes*	Summer visitor		√	√	
*Butastur indicus*	Winter visitor	√			
*Falco peregrinus*	Winter visitor				√
*Falco subbuteo*	Summer visitor		√		

“√” indicates inclusion of the corresponding species-season combination in ensemble modeling; blank cells indicate combinations not included. In total, 22 species-season combinations from 9 representative raptor species were modeled. Species names are given as scientific names only.

## Data Availability

The data that support the findings of this study are available from the first author [Y.Z.] and correspondence author [D.Y.] upon reasonable request.
